# Supplementary material for: Identification of photoperiod-induced specific miRNAs in the adrenal glands of Sunite sheep (Ovis aries)
Source: Front Vet Sci. 2022 Jul 22;9:888207. doi: 10.3389/fvets.2022.888207 (PMC9354845; doi:10.3389/fvets.2022.888207)
Supplement: Supplementary file 1 [file Data_Sheet_1.ZIP › Supplementary file/miRDeep2 file.zip!/miRDeep2 file/Novel_Structure/SPLP42A2/Novel_437.pdf]

Provisional ID	:	NC_019462.2_14718
Score total	:	4
Score for star read(s)	:	3.9
Score for read counts	:	-3.3
Score for mfe	:	2
Score for randfold	:	1.6
Score for cons. seed	:	
Total read count	:	5
Mature read count	:	1
Loop read count	:	0
Star read count	:	4

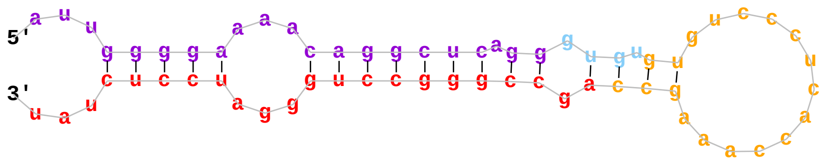

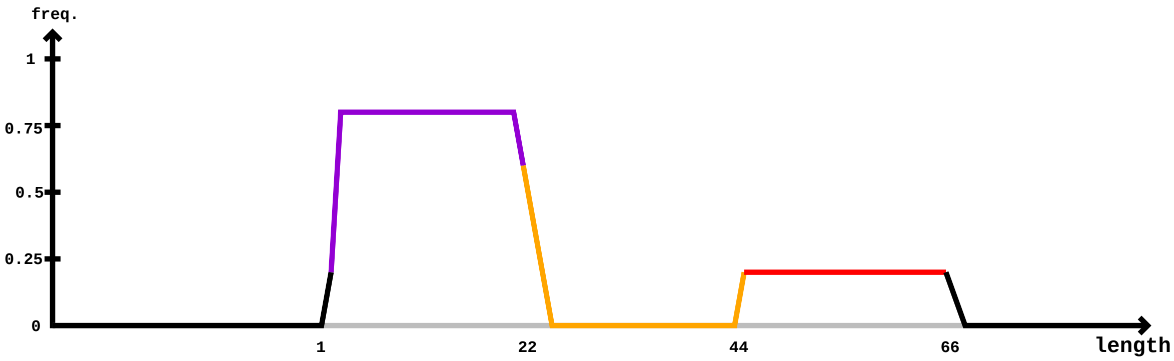


```
                                     Star                                        Mature
5'- gaaauuguggaaaacaggcucagggua auuggggaaaacaggcucagg gugugugucccucaccaaagcc agccgggccugggauccucuau gcccugagguuccugaugac  -3'   obs
    gaaauuguggaaaacaggcucaggguaauuggggaaaacaggcucagggugugugucccucaccaaagccagccgggccugggauccucuaugcccugagguuccugaugac         exp
    ..............((((((((((((...(((((...(((((((.((.((.((.............)))).)))))))))...)))))..)))))))))...)))).....   reads   mm   sample
    ...........................auuggggaaaacaggcucagg................................................................   1       0    seq
    ............................uuggggaaaacaggcucag.................................................................   1       0    seq
    ............................uuggggaaaacaggcucagg................................................................   1       0    seq
    ............................uuggggaaaacaggcucagggu..............................................................   1       0    seq
    ......................................................................agccgggccugggauccucuau....................   1       0    seq
```
